# Effectiveness and Safety of Acupuncture for the Treatment of Alzheimer's Disease: A Systematic Review and Meta-Analysis

**DOI:** 10.3389/fnagi.2020.00098

**Published:** 2020-05-06

**Authors:** Yun-Yun Wang, Shao-Fu Yu, Hong-Yang Xue, Yang Li, Chen Zhao, Ying-Hui Jin

**Affiliations:** ^1^Center for Evidence-Based and Translational Medicine, Zhongnan Hospital of Wuhan University, Wuhan, China; ^2^Center for Evidence-Based and Translational Medicine, Wuhan University, Wuhan, China; ^3^Department of Clinical Pharmacy, The Second People's Hospital of Huaihua, Huaihua, China; ^4^Medical Department of Wuhan University, Wuhan, China; ^5^Institute of Basic Research in Clinical Medicine, China Academy of Chinese Medical Sciences, Beijing, China

**Keywords:** acupuncture, Alzheimer's disease, systematic review, meta-analysis, effectiveness, safety

## Abstract

**Background:** The effects of acupuncture on Alzheimer's disease (AD) outcomes remain controversial. The aim of this review was to evaluate the effectiveness and safety of acupuncture for the treatment of AD.

**Methods:** PubMed, Embase, Web of Science, the Cochrane Central Register of Controlled Trials, Chinese BioMedical Literature Database, VIP Database for Chinese Technical Periodicals, China National Knowledge Infrastructure, and Wanfang Data were searched to identify relevant randomized controlled trials from inception to January 19, 2019. Data were extracted and evaluated by two authors independently. The data analysis was conducted using R (version 3.6.0) and RStudio (version 1.2.1335) software.

**Results:** Thirty trials involving 2,045 patients were included. Acupuncture plus drug therapy may have been more beneficial for general cognitive function in AD patients than drug therapy alone (short-term treatment: MD, mean difference = 1.94, 95% CI: 1.11, 2.77; *p* < 0.01; medium-term treatment: *MD* = 4.41, 95% CI: 1.83, 7.00; *p* < 0.01). People who received acupuncture plus drug therapy attained higher ADL (Activities of Daily Living) scores than patients who received drug therapy alone for medium-term treatment duration (*MD* = −2.14; 95% CI: −3.69, −0.59; *p* < 0.01). However, there is no statistically significant difference in subgroup effect on MMSE (Mini-mental Status Examination) and ADLs (*p* > 0.05) when comparing acupuncture treatment with drug therapy (such as Donepezil hydrochloride, Nimodipine, or Yizhijiannao), or acupuncture plus drug therapy (such as Donepezil hydrochloride, Dangguishaoyaosan, or Jiannaosan) with drug therapy alone. There was also no significant difference in general cognitive function, ADLs, or incidence of adverse events between acupuncture treatment and drug therapy (*p* > 0.05).

**Conclusions:** This review indicates that acupuncture plus drug therapy may have a more beneficial effect for AD patients than drug therapy alone on general cognitive function in the short and medium term and on ADLs in the medium term. Acupuncture alone may not have superior effects compared with drug therapy on global cognitive function, ADLs, and incidence of adverse events. Duration of treatment may not modify the effect of acupuncture in comparison with drug therapy. Additional large-scale and high-quality clinical trials are needed.

## Introduction

Dementia is a progressive global cognitive impairment syndrome. It is estimated that the number of dementia patients worldwide is 35.6 million, and this is expected to double every 20 years and reach 115.4 million by 2050 (World Health Organization, 2013). Alzheimer's disease (AD) is the most common form of dementia, comprising at least 60% of cases; it is characterized by progressive memory deficits, spatial disorientation, and other neuropsychiatric disorders (Herrup, 2011; Thies and Bleiler, 2011). Alzheimer's disease results in a substantial economic burden to patients, society, and the government. The total cost ratio of AD costs to gross domestic product are 1.31 in the Asian Pacific high-income regions, 1.30 in North American high-income regions, 0.97 in Australia, and 0.90–1.29 in Europe (Jia et al., 2018). Pharmacological treatment of Alzheimer's disease focuses on correcting the cholinergic deficiency in the central nervous system with cholinesterase inhibitors. Donepezil, rivastigmine, and galantamine are commonly recommended (Birks, 2005). However, none of these drugs can stop the progression of AD, and their therapeutic effects vary from person to person and are limited to the duration of treatment (Alzheimer's Association, 2017). In addition, adverse events such as nausea, vomiting, and dizziness have been reported to be associated with drug therapy for AD patients (Kobayashi et al., 2016). New effective therapies for AD urgently need to be explored.

Acupuncture can protect neurons from deterioration and promote axonal re-growth in neurodegenerative diseases, such as AD (Li X. et al., 2014). Acupuncture is defined as the placement of solid, sterile, stainless steel needles into specific points on the body, and various techniques are used to stimulate the needles, such as adding a mild electrical current, with the purpose of bringing the patient back to the state of equilibrium postulated to exist prior to illness (Endres et al., 2007). It is a relatively safe treatment with few side effects (Witt et al., 2009) and has been commonly used in clinical practice in China for more than 3,000 years. Recently, there has been increasing interest in acupuncture from both the public and health professionals. According to the result of a survey conducted by the World Federation of Acupuncture and Moxibustion Societies, of the 192 Member States of the United Nations, 178 (93%) have acupuncture practices, and 59 (31%) have acupuncture organizations. Of the various Chinese medicine modalities, acupuncture is the most commonly used worldwide (World Health Organization, 2013).

Previous reviews have revealed some evidence demonstrating that acupuncture plus Chinese herbal medicine was more effective than Western drugs at improving global cognitive function (Zhou et al., 2017). However, studies on the effects of acupuncture compared with conventional Western medicine such as Donepezil, Nimodipine, and Piracetam on general cognitive function have shown contradictory findings and not shown any adequate evidence of any test for the safety of acupuncture (Lee et al., 2009; Zhou et al., 2015; Huang et al., 2019). The real effect of acupuncture alone compared with drug treatment or no treatment, and acupuncture plus drug treatment vs. drug treatment alone on different outcomes such as global cognitive function, the severity of dementia, and skill level in performing the activities of daily living needs further exploration. The use of acupuncture for treating Alzheimer's disease has been increasing in frequency over recent years; it is therefore necessary to re-evaluate its clinical curative effect and safety. The objective of this review was to comprehensively search relevant literature, critically evaluate methodology quality, and summarize and compare the effectiveness and safety of acupuncture therapy administered for different intervention durations in order to help promote the medical treatment of AD in this field.

## Materials and Methods

### Search Strategy and Selection Criteria

PubMed, Embase, Web of Science, the Cochrane Central Register of Controlled Trials, the Chinese BioMedical Literature Database, the VIP Database for Chinese Technical Periodicals, China National Knowledge Infrastructure, and Wanfang Data were comprehensively searched without language restrictions from inception to 19th January 2019. In addition, we also searched the reference lists of all eligible studies and previous systematic reviews for additional relevant studies. The search strategy is presented in Supplementary Material 1 using PubMed as an example.

Two authors independently screened and examined the features of all articles identified using the PICOS (population, interventions, comparators, outcomes, study design) selection criteria. We resolved disagreements by a consensus meeting between the two authors. In cases of duplicate publications, the most recent and complete versions were selected. The PICOS criteria were as follows: (1) Population/participants: people were diagnosed as having AD by definite, clear, and validated diagnostic criteria, including the International Classification of Disease version 9 or 10 (Guy et al., 1984; Statistics NCfH, 1999), the Diagnostic and Statistical Manual of Mental Disorder III, III – R, or IV (American Psychiatric Association, 1994), the National Institute of Neurological and Communicative Disorder and Stroke—Alzheimer's Disease and Related Disorder Association (McKhann et al., 1984; Dubois et al., 2007; Sperling et al., 2011), the advancing research diagnostic criteria for Alzheimer' s disease IWG - 2 criteria (Dubois et al., 2014), and the Diagnosis, Syndrome Differentiation and Efficacy Evaluation Criteria of Senile Dementia (Fu, 1991, 2011); (2) The experimental group used manual acupuncture or electro-acupuncture with or without the same regular therapy as the control group, such as donepezil hydrochloride or rehabilitation training; (3) People in the control group received drug treatments (e.g., donepezil hydrochloride, huperzine, traditional Chinese medicine), sham acupuncture (interventions mimicking true acupuncture/true treatment but deviating in at least one aspect considered important by acupuncture theory, such as skin penetration or correct point location) or non-drug treatment therapy; (4) Outcomes: (1) global cognitive function, which was assessed by using validated scales, such as the Alzheimer's disease assessment scale for cognitive capacity (ADAS-cog), the mini-mental status examination (MMSE), Hasegawa's Dementia Scale (HDS), or the Revised Hasegawa dementia scale (HDS-R); (2) the severity of dementia, which was assessed by using the Clinical Dementia Rating (CDR); (3) skill level on activities of daily living (ADL), which was measured by questionnaires assessing the ability to accomplish activities of daily living; and (4) safety: the number of participants dropping out due to adverse effects (e.g., dizziness, headache, and heart palpitations), and the number of participants reporting adverse effects; and (5) Randomized controlled trials (RCTs).

We excluded studies meeting the following criteria: (1) protocols for an RCT, (2) repeatedly published literature, (3) studies that were unavailable as full text or with vague data, and (4) studies with a lack of usable AD outcomes.

### Data Extraction

An electronic data extraction form was used to extract information on first author, year of publication, sample size, diagnostic criteria, AD categories, age, intervention regime, and methods used in the experimental and control groups (such as acupoints, frequency of acupuncture, and duration of treatment), and outcomes (effectiveness and safety). Data extraction was conducted independently by two investigators, and disagreements were resolved by a consensus meeting between the two authors. For studies with multiple treatment groups, we did not include the irrelevant data from the additional treatment arms.

### Assessment of Risk of Bias in the Included Studies

Two reviewers independently examined the methodological quality of the included RCTs using the criteria described in the Cochrane Handbook for Systematic Reviews of Interventions (http://handbook.cochrane.org/) and then classified the risk of bias as either a low, high, or unclear risk of bias.

### Subgroup Analysis

To achieve our research objectives, we compared the effect of acupuncture vs. drug therapy, acupuncture plus drug therapy vs. drug therapy alone, acupuncture plus non-drug therapy vs. non-drug therapy alone, and acupuncture vs. no treatment. In addition, different treatment sessions in the included RCTs were further considered by grouping the results into the following treatment length time periods: short-term (up to 8 weeks), medium-term (9–12 weeks), and long-term (more than 12 weeks).

### Statistical Analysis

Dichotomous and continuous data were presented as relative risk (RR) and mean difference (MD) with 95% confidence interval (CI), respectively, and standardized mean differences (SMDs) have been used when different scales were applied to measure the same outcome. Heterogeneity among studies was evaluated by the Q statistic and *I*^2^ statistics. A random-effects model was used to pool the data. The *z*-test was performed to evaluate the significance of the pooled results, and a statistically significant difference was defined as *P* < 0.05.

The meta-analysis was conducted using the Meta package with R (version 3.6.0) and RStudio (version 1.2.1335) software. Funnel plots, Egger's tests were used to explore publication bias for each outcome that had >10 studies (Egger et al., 1997; Sterne et al., 2000). Where there was potential publication bias, trim and fill method was used to explore the true effect of the pooled data (Duval and Tweedie, 2000).

## Results

### Study Characteristics

**Figure 1:**
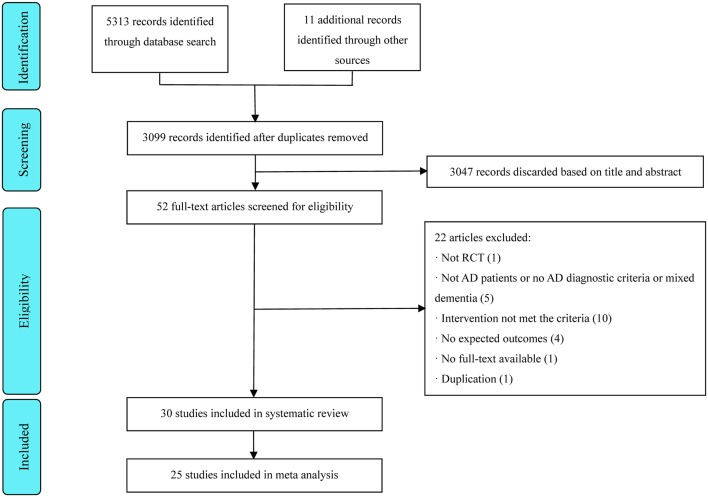
Flow diagram of the study identification and selection process.

**Figure 2:**
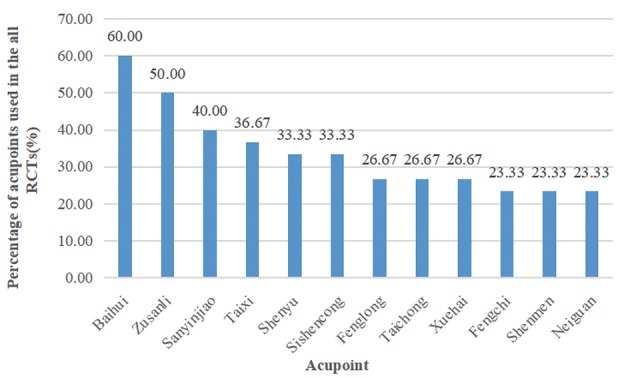
The acupoints commonly used in the all RCTs.

A total of 5,324 articles were identified, and 2,225 duplicates were omitted, leaving 3,099 articles that were screened. Thirty trials involving 2,045 patients published between 1999 and 2019 met all inclusion criteria (Ou et al., 1999; Hou et al., 2000; Dong et al., 2002; Li et al., 2002, 2009; Jiang et al., 2004, 2018; Luo et al., 2006; Zhao et al., 2007; Liu et al., 2008; Hu et al., 2010; Jia et al., 2010, 2017; Zhu et al., 2010; Sun, 2013; Yin et al., 2013; Gu et al., 2014; Lin et al., 2014; Li T. et al., 2014; Wang et al., 2014, 2015; Yan et al., 2014; Lin, 2016; Wei et al., 2016; Guan, 2017; Lou et al., 2017; Peng et al., 2017; Chen et al., 2018; Feng et al., 2019). Preferred Reporting Items for Systematic Reviews and Meta-Analyses flowcharts are shown in Figure 1. The treatment duration ranged from 20 days to 24 weeks. Two studies (Li et al., 2009; Lin, 2016) had a three-arm parallel group design, and three studies (Li et al., 2002; Zhu et al., 2010; Jiang et al., 2018) employed a four-arm parallel group design. The most commonly used acupoints were: Baihui (60%), Zusanli (50%), Sanyinjiao (40%), Taixi (36.67%), Shenyu (33.33%), Sishencong (33.33%), Fenglong (26.67%), Taichong (26.67%), Xuehai (26.67%), Fengchi (23.33%), Shenmen (23.33%), and Neiguan (23.33%) (see Figure 2). Detailed information on the study characteristics is presented in Table 1.

**Table 1:** The characteristic of RCTs included.

**References**	**Sample size**	**Diagnosis criteria**	**AD categories**	**Age, Mean** ± **SD/Range**		**Intervention regimen and methods**		**Outcomes**	
				**Experimental group**	**Control group**	**Experimental group**	**Control group**	**Effectiveness**	**Safety**
Feng et al. (2019)	20/20	OCDAD, NIA-AA	–	68 ± 9	69 ± 7	• Eletroacupuncture: 40 mm needle, dense-sparse wave, (10 Hz/50 Hz, 0.5~5.0 mA); acupoints: Fengfu—(15 mm into the skin, don't retain), Baihui—(15 mm into the skin, retained for 30 min), Shenting—(15 mm into the skin, retained for 30 min), Shangyintang—(15 mm into the skin, retained for 30 min), Temple—(25 mm into the skin, retained for 30 min), Dazhong—(25 mm into the skin, retained for 30 min); one time every 2 days, 3 days every week, last for 12 weeks	• Donepezil hydrochloride 5 mg/tablet, 5 mg/d, taken before bed, last for 12 weeks	MMSE	Incidence of adverse events (e.g., dizziness, headache, palpitation)
Jiang et al. (2018)	20/20/20/20	Guidelines for the diagnosis and treatment of dementia and cognitive impairment in China	Mild to moderate	60~80	60~80	• Jinsanzhen acupuncture combined with Jiannaosan Acupuncture: 25 mm needle; acupoints: Jinsanzhen acupuncture points: Naosanzhen (Naohu and Naokong), Zhisanzhen (Shenting and Benshen), Niesanzhen (two inches above the tip of the ear and one inch behind the ear, one inch before the ear)—(1–1.2 inch flat stab down into the skin, retained for 30 min); once a day, continuously treated for 7 days, rested for 2 days, and then started next round, last for 12 weeks. Jiannaosan: 5 g every time, 2 times/d, last for 12 weeks Jinsanzhen acupuncture Acupoints: same with the former group	• Jiannaosan 5 mg every time, 2 times/d, last for 12 weeks • Donepezil hydrochloride Taken at bed time, 5 mg/d for the first 4 weeks, 10 mg/d for the remaining 8 weeks, last for 12 weeks	MMSE, ADL	–
Chen et al. (2018)	48/48	NIA-AA	–	65~84	67~85	• Acupuncture Acupoints: baihui, renzhong, neiguan, sanyinjiao, xuanzhong, fenglong, taixi; twice a day. The course of treatment was 3 months in both groups • Donepezil hydrochloride 5 mg/time, 1 time/d	• Donepezil hydrochloride The methods of drug treatment was same with experimental group	MMSE, ADL	–
Jia et al. (2017)	35/36	ADRDA, DSM-IV-R	Mild to moderate	75.11 ± 6.53	74.50 ± 6.83	• Acupuncture Acupoints: 1.5 inch needle; acupuntpoints: Danzhong (15 mm into skin), Zhongwan, Waiguan, Zusanli, Qihai (15~25 mm perpendiculary into skin, rotated at small-amplitude and high-frequency with reinforcing method for 30 s), Xuehai (15~25 mm into skin low-frequency reducing method for 30 s), three times/w, last for 12 weeks • Simulant donepezil hydrochloride 5 mg/d, last for 12 weeks	• Donepezil hydrochloride 5 mg/tablet, 5 mg/d for the first 4 weeks, 10 mg/d for the remaining 8 weeks, last for 12 weeks • Sham- acupuncture Acupuncture points: 2 cm away from the acupoint, 0.2 inches into superficial tissue; three times/w, last for 12 weeks	MMSE, ADAS-cog, ADL	Incidence of adverse events (e.g., abnormal blood, urine, stool routine examination, liver and kidney function)
Peng et al. (2017)	25/25	DSM-IV-R, NINDS-AIREN	–	69.4 ± 5.4	69.5 ± 5.3	• Eletroacupuncture A continuous wave, 50 Hz, 2–4 v; acupoints: Shenting, Baihui, Dazhui, Fengfu, Mingmen and Yongquan (0.5~0.8 inch into skin, retained for 25 min), once a day, 10 days a courses, for 3 courses, last for 4 weeks • Huperzine 0.2 mg each dose, 1 dose a day, last for 4 weeks	• Huperzine 0.2 mg each dose, 1 dose a day, last for 4 weeks	MMSE, HDS-R	–
Lou et al. (2017)	60/60	NIA-AA	Mild to moderate	60.56 ± 2.97	61.26 ± 2.83	• Acupuncture Acupoints: Baihui, Sishencong, Fengfu, Yongquan, Taixi, Shenyu, Sanyinjiao, Zusanli, Fenglong; retained for 30 min frequency of treatment: once a day at first, adjusted of syndrome differentiation as treatment time went, last for 24 weeks. • Conventional therapy Donepezil Hydrochloride: 5 mg/tablet, 5 mg/d, for 24 weeks. Acupoint application: heart, kidney, forehead, subcortex, shenmen, Jiaogan; 6 h/d, once a day for 24 weeks	• Conventional therapy The methods of drug treatment was same with experimental group	MoCA, ADL	–
Guan (2017)	30/30	DSM-IV, DSDEE-SD	–	70.5 ± 9.3	69.3 ± 10.2	• Acupuncture Acupoints: frontal midline, parietal midline, temporal front line, temporal posterior line, shenyu, xuanzhong, taixi, zusanli, shuigou, et al.; once a day, 6 times as a course of treatment, a day of rest after one course of treatment; last for 8 weeks • Donepezil, Dirongcuzhi granule Donepezil: take 5 mg before going to bed every day; last for 8 weeks Dirongcuzhi granule: take one dose twice a day; last for 8 weeks	• Donepezil, Dirongcuzhi The methods of drug treatment was same with experimental group	MMSE, ADAS-cog, ADL	–
Wei et al. (2016)	33/33	NINDS-SIANR	–	61~76	60~77	• Acupuncture: acupoints: Baihui, Yongquan, 1–1.5 inches penitrationpenitration into the skin retained for 30 min, once a day, last for 12 weeks • Huperzine 0.2 mg/time, once a day, last for 12 weeks	• Huperzine The methods of drug treatments was similar to the experimental group	MMSE, HDS-R, ADL	Incidence of adverse events (e.g., abdominal distension and abnormal blood routine)
Lin (2016)	30/30/30	DSM-IV, DSDEE-SD	Mild to moderate	50~80	50~80	• Acupuncture Four Shen Needles (about 1.5 inches before and after Baihui), three Brain Needles (ventricle, bilateral brain space), three Zhi Needles (Shenting, bilateral Benshen), and three Temporal Needles (ear tip straight up 2 inches, front and back 1 inch, respectively), Taixi, Taichong, Zusanli, Sanyinjiao, Fenglong, Qihai, and Xuehai; continuous needling for 5 days, rest for 2 days, 4 weeks for 1 course of treatment, continuous observation of three courses of treatment • Acupuncture, Donepezil The methods of acupuncture was the same as mentioned above. Donepezil: 5 mg each time, once a day, 4 weeks as a course of treatment, continuous observation of three courses	• Donepezil The methods of drug treatments was similar to the experimental group	MMSE, ADAS-cog, ADL	–
Wang et al. (2015)	36/36	NINCDS-ADR-DA, DSDEE-SD	Mild to moderate	72.05 ± 3.70	70.31 ± 3.79	• Eletroacupuncture: acupoints: Baihui—(0.8–1.0 inch diagonal stab upward into the skin, retained for 30 min), Dazhui—(0.5–1.0 inch diagonal stab downward into the skin, retained for 30 min), once a day, 6 day every week, last for 12 week	• Donepezil hydrochloride Taken at bed time, 10 mg/tablet, 5 mg/d for the first 4 weeks, 10 mg/d for the remaining 8 weeks, last for 12 weeks	MMSE	–
Gu et al. (2014)	72/69	DSM-IV-R, NINCDS-ADRDA	Mild to moderate	75 ± 7	72 ± 7	• Acupuncture: 40, 50 mm needle; acupoints: Shenting—(13–21 mm plaque into the skin, retained for 30 min), Baihui—(8–13 mm plaque into the skin, retained for 30 min), Fengchi—(26–34 mm plaque into the skin, retained for 30 min), Wangu—(16–32 mm plaque into the skin, retained for 30 min), Danzhong—(5–13 mm plaque into the skin, retained for 30 min), Qihai, Zhongwan—(40 mm straight into the skin, retained for 30 min), Xuehai, Zusanli—(13–26 mm straight into the skin, retained for 30 min) combined with (Tongli, Sanyinjiao, Taixi, Yinlingquan, Tianshu, Fenglong, Taichong), once a day, 6 days every week, 4 weeks for a course, for 4 courses, last for 16 weeks	• Donepezil hydrochloride Taken at bed time, 5.0 mg/tablet, 5 mg/d for the first 4 weeks, 10 mg/d for the remaining 12 weeks. 16 weeks in all	MMSE, ADAS-cog, ADL	Incidence of adverse events (e.g., fainting during acupuncture, sticking needle)
Yan et al. (2014)	20/20	ICD-10	Mild to severe	60~78	60~80	• Acupuncture: 0.5–1.0 inch needle; acupoints: Shenting, Benshen, Sishencong, Shenmen and Taixi matched with Neiguan, Yintang, Taichong, Jianshi, Danzhong, Lianquan, Zhaohai, Zusanli, Yanglingquan, Qihai, Guanyuan, Yinlingquan, Yifeng, Tinggong, Fengchi, Tianzhu, Shuaigu, Tianshu, Hegu, straight stab into the skin, retained for 20 min, once a day, 5 days every week, 4 weeks a course, last for 3 courses, last for 12 weeks	• Donepezil hydrochloride Taken at bed time, 5.0 mg/tablet, 5 mg /d, last for 12 weeks	MMSE	–
Wang et al. (2014)	27/28	DSM-IV	Mild to moderate	70.3 ± 8.0	70.7 ± 9.1	• Acupuncture Acupoints: coronal suture, sagittal suture, lambdoidal suture, frontotemporal sutures—(25–35 mm into the skin at an 15° angle, twirling 200 rpm/min for 1–2 min at the interval of 15 min, retained for 30 min). once a day, 10 days as a course, last for 20 days • Donepezil hydrochloride 5~10 mg/tablets, once a day, 10 days as a course, last for 20 days	• Donepezil hydrochloride Same with experimental group	MMSE, ADAS-cog	–
Zhu (2014)	40/40	CDR, DSDEE-SD	–	71 ± 2	66 ± 2	• Acupuncture Acupoints: Baihui, Sishencong, Zusanli, Taixi, Dazhong, Xuanzhong, Ganyu, Sanyinjiao, Qihai, Geyu. Zusanli, Taixi, Dazhong and Xuanzhong were treated with warm needle moxibustion for 2 strong points, respectively, and were replaced after the needle cooled gradually; once a day, 10 days as a course of treatment, last for 3 months of treatment	• Piracetam 1.4 g each time, 3 times a day, 10 days for 1 a course of treatment, last for 3 months	CDR	–
Lin et al. (2014)	18/18	DSM-IV, NINCDS-ADR-DA	Mild	73.44 ± 3.37	74.21 ± 2.68	• Elecrtoacupuncture: a continuous wave, 80–100 times/min; acupoints: Baihui, Sishencong, Neiguan, Sanyinjiao, 15 mm penetration into the skin, retained for 30 min, once a day, last for 12 weeks	• Donepezil hydrochloride: taken every night before bed, 5 mg/one tablet, 5 mg/every time, last for 12 weeks	MMSE, ADAS-cog, ADL	–
Li T. et al. (2014)	40/40	NINCDS-ADR-DA	Mild to moderate	68.43 ± 7.56	67.32 ± 6.35	• Acupuncture Acupoints: Shenyu, Gaunyuan, Yongquan, Zusanli, Sanyinjiao, Shenmen, Fengchi. 0.3 × 30 mm needle for scalp acupuncture and 0.3 × 40 mm needle for body acupuncture, last for 12 weeks • Rehabilitation training A series of professional training related to skills improvement of life, such as memory, logical thinking, space, and time, live skills, last for 12 weeks	• Rehabilitation training The methods of rehabilitation training was similar to experimental group	MMSE, ADL	–
Yin et al. (2013)	30/30	NINCDS-ADR-DA, DSM-IV-R	–	60~85	60~85	• Acupuncture 1.5 inch needle; acupoints: Qianding to Xuanli, Baihui to Qubin—(30–45 mm into the skin at the interval of 15°, twirling 200 rpm for 1 min, retained for 45 min), once a day, last for 12 weeks • Donepezil hydrochloride 5 mg/d, once a day, last for 12 weeks	• Donepezil hydrochloride The methods of drug treatment was same with the experimental group	MMSE, CDR, ADL	Incidence of adverse events (e.g., abnormal blood, urine, stool routine examination, liver, and kidney function)
Sun (2013)	35/35	DSDEE-SD	–	64.71 ± 9.10	64.4 ± 9.12	• Acupuncture Acupoints: Sishencong, retained for 30 min, last for 4 weeks • Donepezil hydrochloride 5 mg/time, 1 time/d, last for 4 weeks	• Donepezil hydrochloride The methods of drug treatments was similar to experimental group	MMSE, ADL	–
Hu et al. (2010)	40/40	NINCDS-ADR-DA, DSM-IV	–	69.38 ± 6.54	68.08 ± 6.90	• Acupuncture: 1.5 inch needle, acupoints: Danzhong—(0.2–0.5 inch diagonal stab upward into the skin, twirling at high frequency for 30 s), Zhongwan—(1.5 inch straight stab into the skin, twirling at high frequency for 30 s), Qihai—(0.8–1.0 inch straight stab into the skin, twirling at high frequency for 30 s), Xuehai—(1.0–1.5 inch straight stab into the skin, twirling at low frequency sharply for 30 s), Zusanli—(0.5–1.0 inch straight stab into the skin, twirling at high frequency for 30 s), Waiguan—(0.5–1.0 inch straight stab into the skin, twirling at high frequency for 30 s); last for 12 weeks	• No treatment	MMSE, ADL	–
Jia et al. (2010)	25/26	NINCDS-ADR-DA, DSM-IV-R	–	50~90	50~90	Acupuncture: 1.5 inch needle, acupoints: Danzhong—(0.2–0.5 inch diagonal stab upward into the skin, twirling at high frequency for 30 s), Zhongwan—(1.5 inch straight stab into the skin, twirling at high frequency for 30 s), Qihai—(0.8–1.0 inch straight stab into the skin, twirling at high frequency for 30 s), Xuehai—(1.0–1.5 inch straight stab into the skin, twirling at low frequency sharply for 30 s), Zusanli—(0.5–1.0 inch straight stab into the skin, twirling at high frequency for 30 s), Waiguan—(0.5–1.0 inch straight stab into the skin, twirling at high frequency for 30 s); last for 12 weeks	Piracetam: 1.2 g every time, three times/d, last for 12 weeks	MMSE, ADL	–
Zhu et al. (2010)	20/20/20/20	DSM-IV-R	Mild to moderate	60~80	60~80	• Acupuncture 10–75 mm needle, acupoints: Baihui—(0.5–0.8 inch flat stab into the skin, twirling 120 rpm for 1 min, retained for 30 min), Shenyu—(0.5–1.0 inch straight stab into the skin, twirling 120 rpm for 1 min, retained for 30 min), Xuehai—(1–1.5 inch straight into the skin, retained for 30 min), Geyu—(0.5–0.8 inch diagonally stab into the skin, retained for 30 min), once a day, 7 days for a course, last for 8 weeks • Acupuncture combined with Yizhijiannao Yizhijiannao tablets, 5.5 g a time, three times/d, last for 8 weeks	• Yizhijiannao Yizhijiannao tablets, 5.5 g a time, three times/d, last for 8 weeks • Donepezil hydrochloride Taken in the morning, 5 mg/d, last for 8 weeks	MMSE	–
Li et al. (2009)	20/20/20	DSM-IV-R, NINCDS-ADR-DA	Mild to moderate	55~80	55~80	• Acupuncture Acupoints: Shenyu—(1.0 inch straight stab into the skin, retained for 30 min), Geyu—(0.5 inch diagonally stab into the skin, retained for 30 min), Shenmen—(0.5 inch stab into the skin, retained for 30 min), Baihui—(1.0 inch flat stab into the skin); last for 12 weeks • Acupuncture combined with Yizhijiannao The methods of acupuncture was same with acupuncture group. Yizhijiannao tablets: 5.0 g every time, three times/d, last for 12 weeks	• Yizhijiannao The methods of Yizhijiannao was same with experimental group	MMSE, ADAS-cog	–
Liu et al. (2008)	40/40	NINCDS-ADR-DA	Mild to severe	69.16 ± 2.12	68.09 ± 6.24	Acupuncture: 1.5 inch needle, Acupoints: Yingxiang—(one inch above the seal—HT 32, retained for 1 h, manipulate the needle every 10 min), once a day, 5 consecutive days every week, last for 10 weeks	Duxil: 40 mg/tablet, one tablet every time, two times/a day, 5 consecutive days a week, last for 10 weeks	MMSE, HDS-R	–
Zhao et al. (2007)	16/16	DSM-IV-R, DSDEE-SD	–	62~81	62~81	Acupuncture: 40 mm needle, acupoints: Baihui and Dazhui—(0.5 inch stab into the skin, twirling for 5 min, retained for 40 min), last for 8 weeks	Nimodipine: 20 mg every time, three times/d, last for 8 weeks	MMSE, HDS-R, ADL	–
Luo et al. (2006)	48/48	DSM-III, ICD-10	–	50~80	50~80	Elecrtoacupuncture: acupoints: a continuous wave, 2~4 times per second, acupoints: Dazhui, Shenyu, Taixi (bilateral), Zusanli (bilateral), retained for 25 min, 25 days as a course, 3–5 day break between course, last for 75 days	Hydergine: 50 mg/time, three time/d, last for 90 days	MMSE	–
Jiang et al. (2004)	24/20	NINCDS-ADR-DA, DSM-IV-R, DSDEE-SD	–	65.1 ± 6.4	64.3 ± 5.2	Acupuncture: 40 mm needle, acupoints: Baihui, Sishencong, Shenyu, Sanyinjiao, Taixi, Zusanli, and Fenglong—(stab into the skin and twirl, retained for 30 min), Fengchi, Shenmen, Neiguan, Taichong, Geyu, Xuehai—(stab into the skin and twirl); once a day, five times a week, 4 weeks as a course, last for 8 weeks	Huperzine: 50 μg/tablet, 100 μg a time, two times/d, 1 month a course, last for 8 weeks	MMSE, CDR, ADL	–
Dong et al. (2002)	11/10	DSM-III-R	–	46~80	46~80	• Electroacupuncture A continuous wave at first, pulse frequency: 180 times per minute, dense-sparse wave after 15 min; acupoints: Baihui and Dazhui—(Electroacupuncture 180 rpm), Shenyu, Shenmen, Neiguan, Sanyinjiao—(stab and twirl, retained for 40 min), Sishencong and Fengchi—(Electroacupuncture 180 rpm), Taixi, Zusanli, Fenglong, Taichong—(stab and twirl, retained for 40 min), Neiguan, Jianshi, Danzhong, Lianquan, Yanglingquan, Yinlingquan, Yifeng, Tinggong, Yintang, Tianshu, Hegu; once a day, 5 days a week, 4 weeks a course, last for 12 weeks	• Huperzine A Tablets: 100 μg a time, two times a day, 1 month a course, last for 12 weeks	MMSE, ADL	Severity of adverse events (e.g., headache, dizziness, palpitation)
Li et al. (2002)	37/35/18/14	DSM-IV-R	Mild to severe	65 ± 6/67 ± 4	66 ± 4/65 ± 7	• Electroacupuncture A continuous wave, 2–4 Hz, 40 mm needle, acupoints: Baihui, Sishencong, Fengchi, Shenyu—(stab into the skin in the way of Nianzhuanbu), combined with Taichong, Sanyinjiao, Neiguan, Fenglong, Geyu and Xuehai. Once a day, 6 days every week, last for 8 weeks • Electroacupuncture combine with Dangguishaoyaosan The methods of electroacupuncture was same with electroacupuncture group Dangguishaoyaosan: Angelica and Ligusticum chuanxiong (6 g), Paeoniflorin, Poria Cocos, and Rhizoma Atractylodis Macrocephalae (9 g), Alisma orientalis (10 g), decocted in water, one dose/d, for 8 weeks	• Dangguishaoyao san The methods of Dangguishaoyaosan was same as ectroacupuncture combine with Dangguishaoyaosan group • Nimodipine 20–40 mg a time, three times/d, last for 8 weeks	MMSE, HDS, ADL	–
Hou et al. (2000)	30/30	DSDEE-SD	Mild to severe	60~78	61~78	Acupuncture: acupuntpoints: Fengchi, Baihui, Sishencong, Shenmen, Zusanli, Neiguan combined with Taichong, Taixi, Sanyinjiao and Fenglong (retained for 30 min). Once a day, 30 days for a course, last for 2 courses, last for 60 days	250 ml normal saline, Nicholin 0.75 g, intravenous drip, once a day, last for 60 days	HDS-R	–
Ou et al. (1999)	16/14	DSM-IV; ICD-10	Mild to severe	65.53 ± 6.8	64.72 ± 7.6	Electroacupuncture: a continuous wave, 2–4 Hz,1.5 inch needle; acupoints: Baihui, Sishencong and Shenyu (main points), combine with Taichong, Guanyuan, Sanyinjiao and Zusanli—(stab into the skin in the way of twirling for 15 min, retained for 30 min), once a day, 6 days a week, last for 8 weeks	Nimodipine: 20–40 mg a time, 3 times/d, last for 8 weeks	HDS	–

*OCDAD, Operational criteria for the diagnosis of Alzheimer's disease; NIA-AA, National Institute of Aging -Alzheimer's Association; MMSE, Mini-Mental State Examination; ADL, activity of daily living; NINCDS-ADR-DA, The National Institute of Neurological and the Communicative Disorders and Stroke-Alzheimer's Disease and Related Disorders Association; DSM-IV-R, Diagnostic and Statistical Manual of Mental Disorders, fourth edition-revised; ADAS-cog, Alzheimer's disease assessment scale; NINDS-AIREN, National Institute of Neurological Disorders and Stroke-association/Internationale pour la Recherche et l'Enseignement en Neurosciences; HDS-R, Revised Hasegawa Dementia Scale; MoCA, Montreal Cognitive Assessment; DSDEE-SD, Diagnosis, Syndrome Differentiation and Efficacy Evaluation Criteria of Senile Dementia; NINDS-SIANR, The National Institute of Neurological Diseases and Stroke and the Swiss International Association for Neuroscience Research; ICD-10, The International Classification of Diseases; CDR, Clinical Dementia Rating; DSM-III-R, Diagnostic and Statistical Manual of Mental Disorders, third edition-revised*.

### Methodological Quality

The methodological quality of the trials was generally unsatisfactory. The main characteristics of the trials are displayed in Table 2. Overall, 13 trials appropriately described the methods of randomization by means of a random number table or SAS software (Liu et al., 2008; Gu et al., 2014; Li T. et al., 2014; Wang et al., 2014, 2015; Zhu, 2014; Lin, 2016; Guan, 2017; Jia et al., 2017; Lou et al., 2017; Peng et al., 2017; Chen et al., 2018; Feng et al., 2019). One RCT used a semi-randomization method, and participants were assigned by order of investigation (Hu et al., 2010). More than 90% did not report details of allocation concealment or blinding. Only one RCT conducted sham-acupuncture in the control group, which is regarded as blinding the therapist and participants (Jia et al., 2017). None of the studies demonstrated attrition bias, reporting bias, or other biases.

**Table 2:** Quality assessment of methodology of included studies.

**References**	**Sequence generation**	**Allocation concealment**	**Blinding**		**Incomplete outcome data**	**Selective outcome reporting**	**Other sources of bias**
			**Therapist and participants**	**Outcome assessors**			
Feng et al. (2019)	Low risk	Low risk	Unclear	Unclear	Low risk	Low risk	Low risk
Jiang et al. (2018)	Unclear	Unclear	Unclear	Unclear	Low risk	Low risk	Low risk
Chen et al. (2018)	Low risk	Unclear	Unclear	Unclear	Unclear	Low risk	Low risk
Jia et al. (2017)	Low risk	Unclear	Low risk	Low risk	Low risk	Low risk	Low risk
Peng et al. (2017)	Low risk	Unclear	Unclear	Unclear	Low risk	Low risk	Low risk
Lou et al. (2017)	Low risk	Unclear	Unclear	Unclear	Low risk	Low risk	Low risk
Guan (2017)	Low risk	Unclear	Unclear	Unclear	Low risk	High risk	Low risk
Wei et al. (2016)	Unclear	Unclear	Unclear	Unclear	Low risk	Low risk	Low risk
Lin (2016)	Low risk	Unclear	Unclear	Unclear	Unclear	High risk	Low risk
Wang et al. (2015)	Low risk	Unclear	Unclear	Unclear	Low risk	Low risk	Low risk
Gu et al. (2014)	Low risk	Unclear	Unclear	Unclear	Low risk	Low risk	Low risk
Yan et al. (2014)	Unclear	Unclear	Unclear	Unclear	Low risk	Low risk	Low risk
Wang et al. (2014)	Low risk	Unclear	Unclear	Low risk	Low risk	Low risk	Low risk
Zhu (2014)	Low risk	Unclear	Unclear	Unclear	Low risk	Low risk	Low risk
Lin et al. (2014)	Unclear	Unclear	Unclear	Unclear	Low risk	Low risk	Low risk
Li T. et al. (2014)	Low risk	Unclear	Unclear	Unclear	Low risk	Low risk	Low risk
Yin et al. (2013)	Unclear	Unclear	Unclear	Unclear	Low risk	Low risk	Low risk
Sun (2013)	Unclear	Unclear	Unclear	Unclear	Low risk	Low risk	Low risk
Hu et al. (2010)	Low risk	Unclear	Unclear	Unclear	Low risk	Low risk	Low risk
Jia et al. (2010)	Unclear	Unclear	Unclear	Unclear	Low risk	Low risk	Low risk
Zhu et al. (2010)	Unclear	Unclear	Unclear	Unclear	Low risk	Low risk	Low risk
Li et al. (2009)	Unclear	Unclear	Unclear	Unclear	Low risk	Low risk	Low risk
Liu et al. (2008)	Low risk	Unclear	Unclear	Unclear	Low risk	Low risk	Low risk
Zhao et al. (2007)	Unclear	Unclear	Unclear	Unclear	Low risk	Low risk	Low risk
Luo et al. (2006)	Unclear	Unclear	Unclear	Unclear	Low risk	Low risk	Low risk
Jiang et al. (2004)	Unclear	Unclear	Unclear	Unclear	Low risk	Low risk	Low risk
Dong et al. (2002)	Unclear	Unclear	Unclear	Unclear	Low risk	Low risk	Low risk
Li et al. (2002)	Unclear	Unclear	Unclear	Unclear	Low risk	Low risk	Low risk
Hou et al. (2000)	Unclear	Unclear	Unclear	Unclear	Low risk	Low risk	Low risk
Ou et al. (1999)	Unclear	Unclear	Unclear	Unclear	Low risk	Low risk	Low risk

### Global Cognitive Function-MMSE

#### Acupuncture vs. Drug Therapy

**Figure 3:**
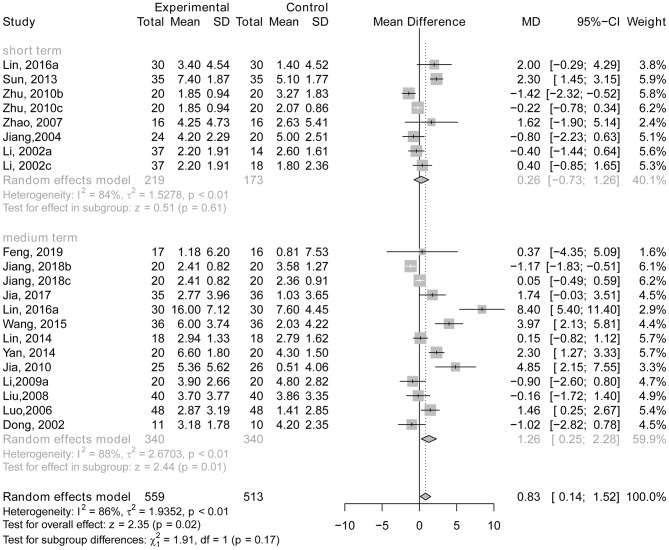
The forest plot of MMSE of acupuncture vs. drug therapy. Lin (2016a), acupuncture vs. Donepezil; Zhu (2010b), acupuncture vs. Donepezil hydrochloride; Zhu (2010c), acupuncture vs. Yizhijiannao; Li (2002a), electroacupuncture vs. Nimodipine; Li (2002c), electroacupuncture vs. Dangguishaoyaosan; Jiang (2018b), acupuncture vs. Donepezil hydrochloride; Jiang (2018c), acupuncture vs. Jiannaosan; Li (2009a), acupuncture vs. Yizhijiannao.

**Figure 4:**
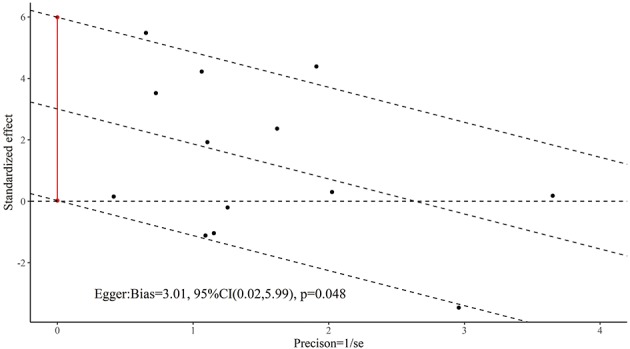
The Egger test result for general cognitive function of acupuncture vs. drug therapy in the medium term.

**Figure 5:**
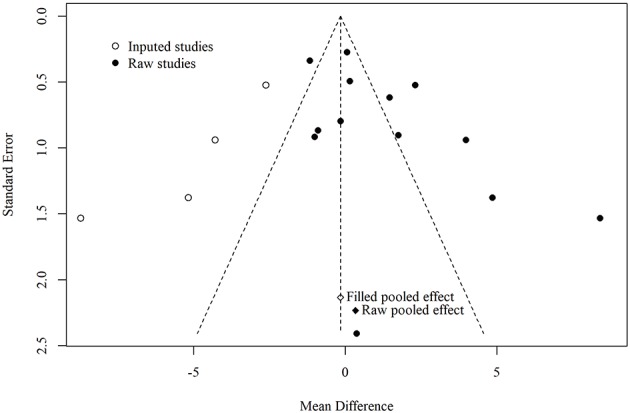
The funnel plot for general cognitive function of acupuncture vs. drug therapy in the medium.

Six trials (Li et al., 2002; Jiang et al., 2004; Zhao et al., 2007; Zhu et al., 2010; Sun, 2013; Lin, 2016) reported data using the MMSE to compare the effects of acupuncture with drug therapy following short-term treatment, and 12 RCTs (Dong et al., 2002; Luo et al., 2006; Liu et al., 2008; Li et al., 2009; Jia et al., 2010, 2017; Lin et al., 2014; Yan et al., 2014; Wang et al., 2015; Lin, 2016; Jiang et al., 2018; Feng et al., 2019) reported data following medium-term treatment. There was no statistically significant difference between the experimental groups and the control groups with short-term treatment (*MD* = 0.26; 95% CI: −0.73, 1.26; *p* = 0.61). Although there was a statistically significant difference with medium-term treatment (*MD* = 1.26; 95% CI: 0.25, 2.28; *p* = 0.01; see Figure 3), as Figures 4, 5 show, there is evidence of a publication bias with regard to the MMSE results when comparing the effect of acupuncture with drug therapy in the medium term (*p* = 0.048 for Egger's test, asymmetric funnel plot). After using trim and fill method, the results revealed that there was no statistically significant difference between the two groups (Figure 5). In addition, only one study demonstrated that the MMSE score measured in the experimental group was not higher than that in the control group in the studies with long-term treatment (*p* > 0.05; Gu et al., 2014).

The test for differences between short-term treatment and medium-term treatment suggested that there is no statistically significant subgroup effect (*p* = 0.17), indicating that duration of treatment may not modify the effect of acupuncture in comparison to drug therapy (see Figure 3).

#### Acupuncture Plus Drug Therapy vs. Drug Therapy Alone

**Figure 6:**
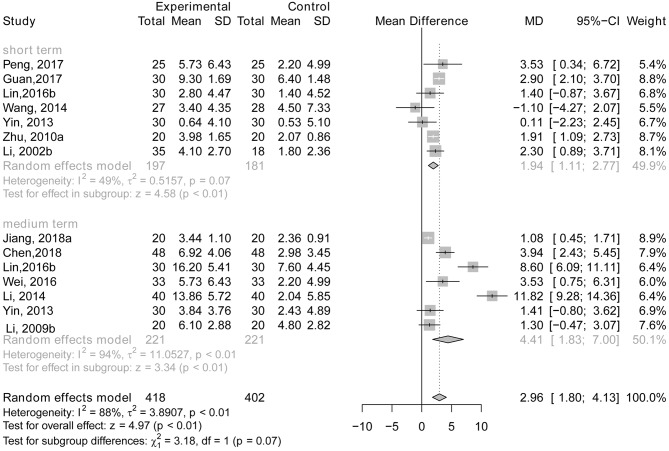
The forest plot of MMSE of acupuncture plus drug therapy vs. drug therapy alone. Lin (2016b), acupuncture plus Donepezil vs. Donepezil; Zhu (2010a), acupuncture plus Yizhijiannao vs. Yizhijiannao; Jiang (2018a), acupuncture plus Jiannaosan vs. Jiannaosan; Li (2009b), acupuncture plus Yizhijiannao vs. Yizhijiannao.

There was a significant difference between the experimental group and the control group in the studies with short-term treatment (*MD* = 1.94, 95% CI: 1.11, 2.77; *p* < 0.01; Li et al., 2002; Zhu et al., 2010; Yin et al., 2013; Wang et al., 2014; Lin, 2016; Guan, 2017; Peng et al., 2017) and medium-term treatment (*MD* = 4.41, 95% CI: 1.83, 7.00; *p* < 0.01; Li et al., 2009; Yin et al., 2013; Li T. et al., 2014; Lin, 2016; Wei et al., 2016; Chen et al., 2018; Jiang et al., 2018), indicating that AD patients receiving acupuncture plus drug therapy performed better on general cognitive function than those who only received drug therapy (see Figure 6). There were no studies reporting long-term treatment that showed any difference in efficacy between the two groups using MMSE.

There is no statistically significant subgroup effect between short-term treatment and medium-term treatment (*p* = 0.07), indicating that duration of treatment may not modify the effect of acupuncture plus drug therapy compared with drug therapy alone (see Figure 6).

#### Acupuncture Plus Non-drug Therapy vs. Non-drug Therapy Alone

One study (Li T. et al., 2014) demonstrated that treatment with acupuncture plus rehabilitation training is more beneficial for patients' global cognitive function than treatment with rehabilitation training alone (MMSE score after treatment: the experimental group, 21.38 ± 6.39; the control group: 19.72 ± 5.25; *p* < 0.05). No data were provided on the effect on the two groups with short-term or long-term treatment.

#### Acupuncture vs. No Treatment Control

One RCT (Hu et al., 2010) demonstrated that there was no significant difference in global cognitive function between the two groups after short term treatment (after treatment: the experimental group, 14.80 ± 5.53; the control group: 13.12 ± 4.98; *p* > 0.05). However, people in the experimental group obtained higher MMSE scores than those in the control group (after treatment: the experimental group, 16.52 ± 6.19; the control group: 12.78 ± 4.68; *p* < 0.05), suggesting that acupuncture therapy may be beneficial for individuals with AD after medium term treatment.

### Global Cognitive Function-ADAS-cog

#### Acupuncture vs. Drug Therapy

No studies focused on the therapeutic effect of acupuncture compared with drug therapy in the short term.

**Figure 7:**
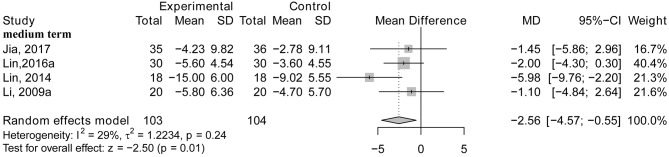
The forest plot of ADAS-cog of acupuncture vs. drug therapy. Lin (2016a), acupuncture vs. Donepezil; Li (2009a), acupuncture vs. Yizhijiannao.

The results from four RCTs using medium-term treatment length indicated that AD patients who received acupuncture therapy did not attain better improvements in general cognitive function than those who received drug treatments (*MD* = −2.56; 95% CI: −4.57, −0.55; *p* = 0.01; Li et al., 2009; Lin et al., 2014; Lin, 2016; Jia et al., 2017; see Figure 7). One study demonstrated the effect of acupuncture vs. drug therapy on general cognitive function using the ADAS-cog with long term treatment and found that there was no significant effect between the two groups (*p* > 0.05; Gu et al., 2014).

#### Acupuncture Plus Drug Therapy vs. Drug Therapy Alone

**Figure 8:**
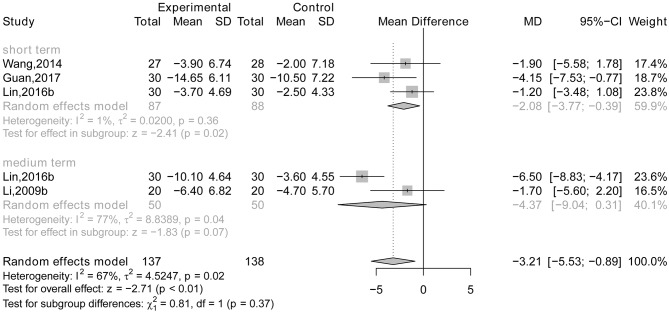
The forest plot of ADAS-cog of acupuncture plus drug therapy vs. drug therapy alone. Lin (2016b), acupuncture plus Donepezil vs. Donepezil; Li (2009b), acupuncture plus Yizhijiannao vs. Yizhijiannao.

There is a statistically significant difference between short-term treatment (*MD* = −2.08, 95% CI: −3.77, −0.39; *P* = 0.02; Wang et al., 2014; Lin, 2016; Guan, 2017) and medium-term treatment (*MD* = −4.37, 95% CI: −9.04, 0.31; *p* = 0.07; Li et al., 2009; Lin, 2016; see Figure 8). However, there were no data reporting a difference in efficacy between the two groups using the ADAS-cog after long-term treatment.

There is no statistically significant subgroup effect between short-term treatment and medium-term treatment (*p* = 0.37), indicating that duration of treatment may not modify the effect of acupuncture plus drug therapy compared with drug therapy alone (see Figure 8).

### Global Cognitive Function-HDS

#### Acupuncture vs. Drug Therapy

**Figure 9:**
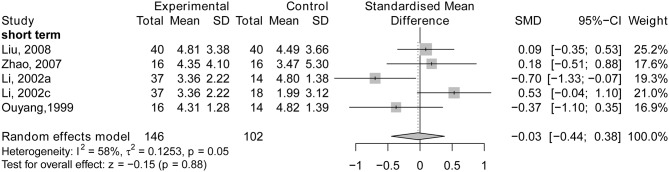
The forest plot of HDS of acupuncture vs. drug therapy. Li (2002a), acupuncture vs. Nimodipine; Li (2002c), acupuncture vs. Dangguishaoyaosan.

Four short-term trials (Ou et al., 1999; Li et al., 2002; Zhao et al., 2007; Liu et al., 2008) demonstrated that no significant difference was found between the experimental group and control group (SMD = −0.03; 95% CI: −0.44, 0.38; *p* = 0.88; see Figure 9). One study also found that there was no statistically significant difference between the two groups after medium-term treatment (*p* > 0.05; Hou et al., 2000). No RCTs were identified showing the effects of long-term treatment.

#### Acupuncture Plus Drug Therapy vs. Drug Therapy Alone

**Figure 10:**
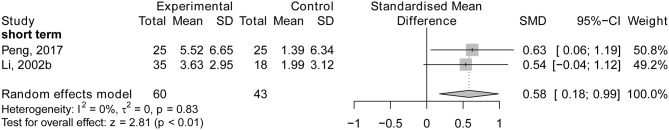
The forest plot of HDS of acupuncture plus drug therapy vs. drug therapy alone. Li (2002b), acupuncture plus Dangguishaoyaosan vs. Dangguishaoyaosan.

There was a statistically significant difference between the acupuncture plus drug therapy and the drug therapy alone after short-term treatment (SMD = 0.58, 95% CI: 0.18, 0.99; *p* < 0.01; Li et al., 2002; Peng et al., 2017; see Figure 10). One study focused on the difference in the efficacy for the two groups after medium-term treatment and found that AD patients in the experimental group showed better improvements in general cognitive function than those in the control group (after treatment: the experimental group, 17.97 ± 6.19; the control group: 18.88 ± 6.35; *p* < 0.05; Wei et al., 2016). No long-term treatment studies were identified.

### Global Cognitive Function-MoCA

#### Acupuncture Plus Drug Therapy vs. Drug Therapy Alone

One study (Lou et al., 2017) demonstrated that acupuncture combined with drug therapy was more beneficial for general cognitive function among AD patients than drug therapy alone after long-term treatment (after treatment: the experimental group, 26.52 ± 0.69; the control group: 21.26 ± 0.28; *p* < 0.05). No studies were identified exploring short-term or medium-term treatment.

### Severity of Dementia-CDR

#### Acupuncture vs. Drug Therapy

One trial (Jiang et al., 2004) evaluated the therapeutic effect of acupuncture compared with huperzine in the short term and found no significant difference between the two groups after treatment (CDR score, the experimental group: 2.10 ± 0.50, the control group: 2.20 ± 0.40; *p* > 0.05). No trials were found exploring the different effects for the two groups after medium- or long-term treatment.

#### Acupuncture Plus Drug Therapy vs. Drug Therapy Alone

One RCT (Yin et al., 2013) found that there was no significant difference between the experimental group and the control group after short-term treatment (*p* > 0.05), but the severity of dementia in AD patients in the experimental group was reduced compared with that in the control group after medium-term treatment (CDR score, the experimental group: 1.50 ± 0.57, the control group: 1.83 ± 0.70, *p* < 0.05). No studies of long-term treatment were identified.

### Skill Level on Activities of Daily Living

#### Acupuncture vs. Drug Therapy

**Figure 11:**
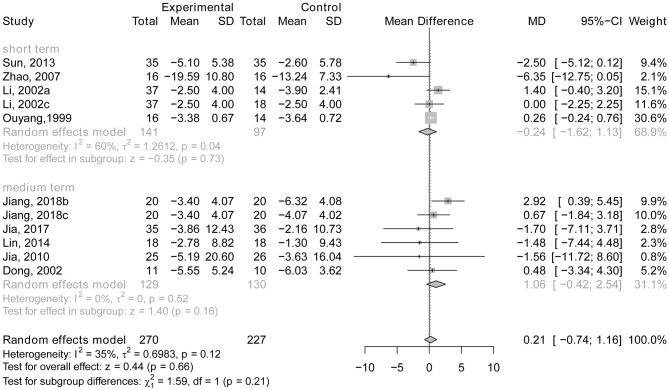
The forest plot of ADL of acupuncture vs. drug therapy. Li (2002a), acupuncture vs. Nimodipine; Li (2002c), acupuncture vs. Dangguishaoyaosan; Jiang (2018b), acupuncture vs. Donepezil hydrochloride; Jiang (2018c), acupuncture vs. Jiannaosan.

Four RCTs concentrated on the effect of acupuncture compared with drug therapy on ADLs after short-term treatment (Ou et al., 1999; Li et al., 2002; Zhao et al., 2007; Sun, 2013) and five trials (Dong et al., 2002; Jia et al., 2010, 2017; Lin et al., 2014; Jiang et al., 2018) concentrated on the effect after medium-term treatment. The MD was −0.24 (95% CI: −1.62, 1.13; *p* = 0.73) in the studies with short-term treatment and (*MD* = 1.06; 95% CI: −0.42, 2.54; *p* = 0.16) in the studies with medium-term treatment, indicating that individuals with AD who receive acupuncture therapy do not perform better in ADLs than those who received drug treatment (see Figure 11). In addition, only one trial (Gu et al., 2014) focused on the effect of long-term acupuncture compared with drug therapy on ADLs. A slightly significant effect was found between the two groups, and the general cognitive function of AD patients receiving acupuncture therapy performed better than those in the control group (*p* < 0.05).

There is no statistically significant subgroup effect (*p* = 0.21), indicating that duration of treatment may not modify the effect of acupuncture in comparison with drug therapy (see Figure 11).

#### Acupuncture Plus Drug Therapy vs. Drug Therapy Alone

**Figure 12:**
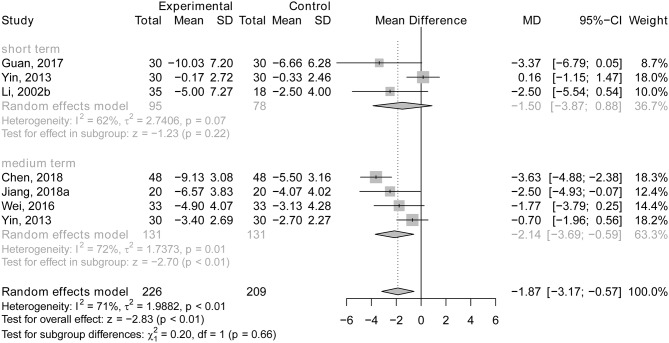
The forest plot of ADL of acupuncture plus drug therapy vs. drug therapy alone. Li (2002b), acupuncture plus Dangguishaoyaosan vs. Dangguishaoyaosan; Jiang et al. (2018), acupuncture plus Jiannaosan vs. Jiannaosan.

There was no statistically significant difference between the experimental group and control group after short-term therapy (*MD* = −1.50; 95% CI: −3.87, 0.88; *p* = 0.22; Li et al., 2002; Yin et al., 2013; Guan, 2017; see Figure 12). However, people in the experimental group performed better on ADLs than those in the control group after medium-term treatment (*MD* = −2.14; 95% CI: −3.69, −0.59; *p* < 0.01; Yin et al., 2013; Wei et al., 2016; Chen et al., 2018; Jiang et al., 2018; see Figure 12). One study (Lou et al., 2017) demonstrated that acupuncture combined with drug therapy had a more beneficial effect on ADLs in AD patients than drug therapy alone after long-term treatment (after treatment: the experimental group, 20.15 ± 1.08; the control group: 25.31 ± 1.69; *p* < 0.05).

There is no statistically significant subgroup effect (*p* = 0.66), indicating that duration of treatment may not modify the effect of acupuncture in comparison with drug therapy (see Figure 12).

#### Acupuncture Plus Non-drug Therapy vs. Non-drug Therapy Alone

One study (Li T. et al., 2014) found that acupuncture plus rehabilitation training is more beneficial for patients' skill level on the ADL scale than therapy consisting of rehabilitation training alone (after medium term treatment: the experimental group, 78.31 ± 20.45; the control group: 58.13±18.72; *p* < 0.05). No studies provided data on the effects for the two groups after short-term or long-term treatment.

#### Acupuncture vs. No Treatment Control

One RCT (Hu et al., 2010) found that there was no significant difference in participants' ADL skill level between the two groups after short term therapy (after treatment: the experimental group, 50.12 ± 18.71; the control group: 54.25 ± 18.78; *p* > 0.05). However, compared with the control group, acupuncture therapy may have a more beneficial effect for individuals with AD on ADL skill level after medium term treatment (after treatment: the experimental group, 45.68 ± 17.12; the control group: 54.50 ± 18.40; *p* < 0.05).

### Safety

#### Acupuncture vs. Drug Therapy

**Figure 13:**
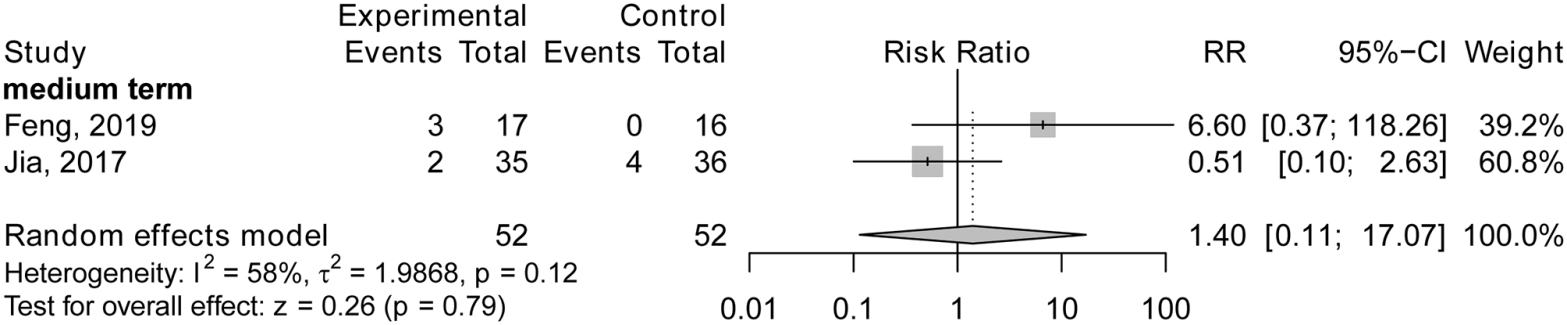
The forest plot of incidence of adverse events of acupuncture vs. drug therapy.

No RCTs studied the difference between acupuncture therapy and drug treatment on the incidence of adverse events after short-term treatment. Nevertheless, two trials (Jia et al., 2017; Feng et al., 2019) found that there was no statistically significant difference between the two groups on the incidence of adverse events after medium-term treatment (RR = 1.40; 95% CI: 0.11, 17.07; *p* = 0.79; see Figure 13). One study (Gu et al., 2014) found that the incidence of adverse events in the experimental group decreased compared with the control group following long-term treatment (the experimental group: 0%, the control group: 13.04%, *p* < 0.05).

#### Acupuncture Plus Drug Therapy vs. Drug Therapy Alone

No trials focused on the incidence of adverse events between the experimental group and control group following short-term or long-term treatment. Only one study reported that there was no statistically significant difference between the two groups following medium-term treatment (*p* > 0.05; Wei et al., 2016).

## Discussion

30 RCTs (2,045 patients) were included in this review. The main finding of our review suggests acupuncture plus drug therapy may be more beneficial for AD patients than drug therapy alone in the areas of general cognitive function in the short term and medium term, and ADL skills in the medium term. However, acupuncture alone may not have superior effects when compared with drug therapy on general cognitive function, ADL skills, or incidence of adverse events. Duration of treatment may not modify the effect of acupuncture in comparison with drug therapy.

The MMSE scale is the most commonly used measurement tool for evaluating general cognitive function (Folstein et al., 1975). A total of 23 RCTs (Dong et al., 2002; Li et al., 2002, 2009; Jiang et al., 2004, 2018; Luo et al., 2006; Zhao et al., 2007; Liu et al., 2008; Hu et al., 2010; Jia et al., 2010, 2017; Zhu et al., 2010; Sun, 2013; Yin et al., 2013; Lin et al., 2014; Li T. et al., 2014; Wang et al., 2014, 2015; Yan et al., 2014; Wei et al., 2016; Peng et al., 2017; Feng et al., 2019) in our review explored the therapeutic effects by using MMSE scores. The majority of trials offered donepezil hydrochloride and huperzine to AD patients in the control groups. The findings revealed that people who received acupuncture therapy did not attain higher scores than patients who received drug treatments, which is consistent with Lee's findings (Lee et al., 2009) but contrary to Zhou's findings (Zhou et al., 2015) based on 6 trials (359 participants). The small sample size in Zhou's research may limit the power of the evidence. The confidence interval of the pooled effect was very close to the invalid line in the forest plot of the effect of acupuncture vs. drugs on the MMSE score. When the sample size increased, the pooled results of the study may have changed, as in our review. In addition, we found that acupuncture combined with drug therapy may have been more beneficial for general cognitive function in AD patients than drug therapy alone; individuals with AD in the experimental group attained higher MMSE scores than those in the control group, which is similar to Zhou's findings (Zhou et al., 2015). Moreover, the results of subgroup difference may fail to support the positive effect that durations of therapy may have on general cognitive function when comparing acupuncture treatment with drug therapy, or acupuncture plus drug therapy with drug therapy alone on MMSE. The reliability of the results was problematic due to unexplained heterogeneity. It is also essential to consider that the methodological quality of the RCTs in our review was low. Random sequence generation, allocation concealment, blinding of participants and personnel, and blinding of outcome assessments are the key elements of RCT methodology quality. Thirteen trials did not provide detailed information on the items mentioned above (Dong et al., 2002; Li et al., 2002, 2009; Jiang et al., 2004, 2018; Luo et al., 2006; Zhao et al., 2007; Jia et al., 2010; Zhu et al., 2010; Sun, 2013; Lin et al., 2014; Yan et al., 2014; Wei et al., 2016). Eight RCTs generated random sequences using a random number table and statistical software (Liu et al., 2008; Hu et al., 2010; Li T. et al., 2014; Wang et al., 2014, 2015; Jia et al., 2017; Peng et al., 2017; Feng et al., 2019). Only one RCT concealed the allocation scheme using closed envelopes (Feng et al., 2019), and one trial added simulated donepezil hydrochloride in the experimental group and sham acupuncture in the control group in an attempt to blind the AD patients and study personnel (Jia et al., 2017). The outcome assessment in two RCTs was performed by a person who was not involved in the treatment (Wang et al., 2014;Jia et al., 2017).

With the progressive deterioration of cognitive function in AD patients, their ability to perform activities of daily living is also significantly affected. Eleven trials focused on the effect of acupuncture on ADL performance in AD patients (Ou et al., 1999; Dong et al., 2002; Li et al., 2002; Zhao et al., 2007; Jia et al., 2010, 2017; Sun, 2013; Yin et al., 2013; Lin et al., 2014; Wei et al., 2016; Jiang et al., 2018). We found that acupuncture has no obvious advantages over drug treatment on ADL performance. But people who received acupuncture plus drug therapy attained higher scores on the ADL scale than patients who received drug therapy alone after 9–12 weeks of treatment. Similar findings were shown in Zhou's study (Zhou et al., 2015) based on 2 RCTs with 12 weeks of treatment (Zou and Yang, 2011; Jin, 2014). We did not find that duration of treatment played a positive role in exploring the real effect on ADLs when comparing acupuncture treatment with drug therapy, or acupuncture plus drug therapy with drug therapy alone. However, the low methodological quality of the RCTs included in our review may affect the reliability of the results. For example, 10 RCTs provided no information on the methods of random sequence generation, allocation concealment, blinding of participants and personnel, or blinding of outcome assessment (Ou et al., 1999; Dong et al., 2002; Li et al., 2002; Zhao et al., 2007; Jia et al., 2010; Sun, 2013; Yin et al., 2013; Lin et al., 2014; Wei et al., 2016; Jiang et al., 2018). Only one RCT minimized the risk of selection bias, performance bias, and detection bias to some extent by means of rigorous random sequence generation as well as blinding of the participants, personnel, and outcome assessors (Jia et al., 2017).

Additionally, there were other metrics used to evaluate the improvements among AD individuals, including the MoCA, ADAS-cog, HDS, CDR, and incidence of adverse events, which were limited by the sample sizes and the small number of studies. The results indicated that acupuncture may not be more effective than drug treatment or enhance the therapeutic effect of drug treatment but may at least have some similar effects compared with drug treatment for AD patients. We were not able to draw reliable conclusions on the effect of acupuncture compared with other control treatment types (non-drug therapy, no treatment) based on an inadequate number of RCTs.

Acupuncture therapy may modulate neuron synaptic plasticity to relieve cognitive impairment by means of insertion at and stimulation of certain points (Han et al., 2018; Zheng et al., 2018). The possible mechanisms of its therapeutic effect for AD are as follows. First, the imbalance between the production and clearance of β-amyloid in the brain increases the level of Aβ, resulting in neurovascular toxicity, hippocampal dysfunction, and cell death (Zhou et al., 2019). Acupuncture may reduce the expression of β-amyloid 42-positive cells in the hippocampus and improve the deposition of β-amyloid proteins (Jiang et al., 2016). Second, the density of neurofibrillary tangles in the brain is positively correlated with the degree of cognitive impairment in AD patients (Gómez-Isla et al., 1997). Acupuncture can regulate the p38MAPK pathway, reduce the expression of phosphorylated Tau protein in the hippocampus of AD rats, and enhance learning and memory ability. Acupuncture has also been correlated with inhibition of the p38MAPK signaling pathway in the hippocampus and downregulation of the overphosphorylation of Tau protein (Zhang et al., 2017). Third, acupuncture for AD reduces β-amyloid deposits and has beneficial effects on cognitive dysfunction, with a focus on the stimulation of neurogenesis and increased BDNF (brain-derived neurotrophic factor) expression in the brain (Shin et al., 2017). Fourth, acupuncture at either a single acupoint or a combination of acupoints can play a role in the central cholinergic nervous system, thus promoting the transmission function of the central nervous system and improving cognitive function (Luo B. et al., 2017; Luo B. H. et al., 2017). In general, acupuncture may have a positive effect on AD patients in various ways.

Nevertheless, this review has a number of limitations to be considered. First, the methodological and reporting qualities of the included RCTs were unsatisfactory. Descriptions of the randomization procedures, allocation concealment, and blinding of participants, study personnel, and outcome assessors were problematic in some of the studies. In addition, the success of acupuncture treatment in this context depends on several factors, such as the selection of acupoints, timing of the acupuncture, the duration of the sessions, and the selection of the stimulation technique; these factors differed between the included trials. Thus, high-quality and large-scale RCTs should be performed in the future. The RCTs should investigate the characteristics of acupuncture that are essential for its effectiveness (i.e., mode of administration, pattern of stimulation, choice of needles, number of sessions) to determine the potential relevance of such characteristics to the effectiveness of acupuncture for AD.

And they can focus on the comparison of acupuncture plus drug therapy vs. acupuncture in order to further understand the effect of acupuncture for AD patients.

## Conclusion

Acupuncture plus drug therapy may have a more beneficial effect for AD patients than drug therapy alone on general cognitive function in the short term and medium term and on ADL skills in the medium term. However, acupuncture alone may not have superior effects as compared with drug therapy on global cognitive function, ADLs, or incidence of adverse events. What's more, duration of treatment may not modify the effect of acupuncture in comparison with drug therapy. Additional large-scale and high-quality RCTs will be needed to draw a more definitive conclusion.

## Author Contributions

Y-YW, S-FY, and Y-HJ designed the review and determined inclusion eligibility of RCTs. H-YX, YL, and CZ carried out literature searches, study selection, and data extraction. Y-YW, S-FY, and Y-HJ assessed the quality of studies, contributed to the analysis, and interpretation of the data. Y-YW wrote the manuscript. Y-HJ revised the manuscript. All authors read and approved the final manuscript.

## Conflict of Interest

The authors declare that the research was conducted in the absence of any commercial or financial relationships that could be construed as a potential conflict of interest.

## Supplementary Material

Supplementary Data Sheet 1 The search strategy on PubMed.
