# Supplementary figures and images for: Effectiveness and Safety of Acupuncture for the Treatment of Alzheimer's Disease: A Systematic Review and Meta-Analysis
Source: Front Aging Neurosci. 2020 May 6;12:98. doi: 10.3389/fnagi.2020.00098 (PMC7218057; doi:10.3389/fnagi.2020.00098)

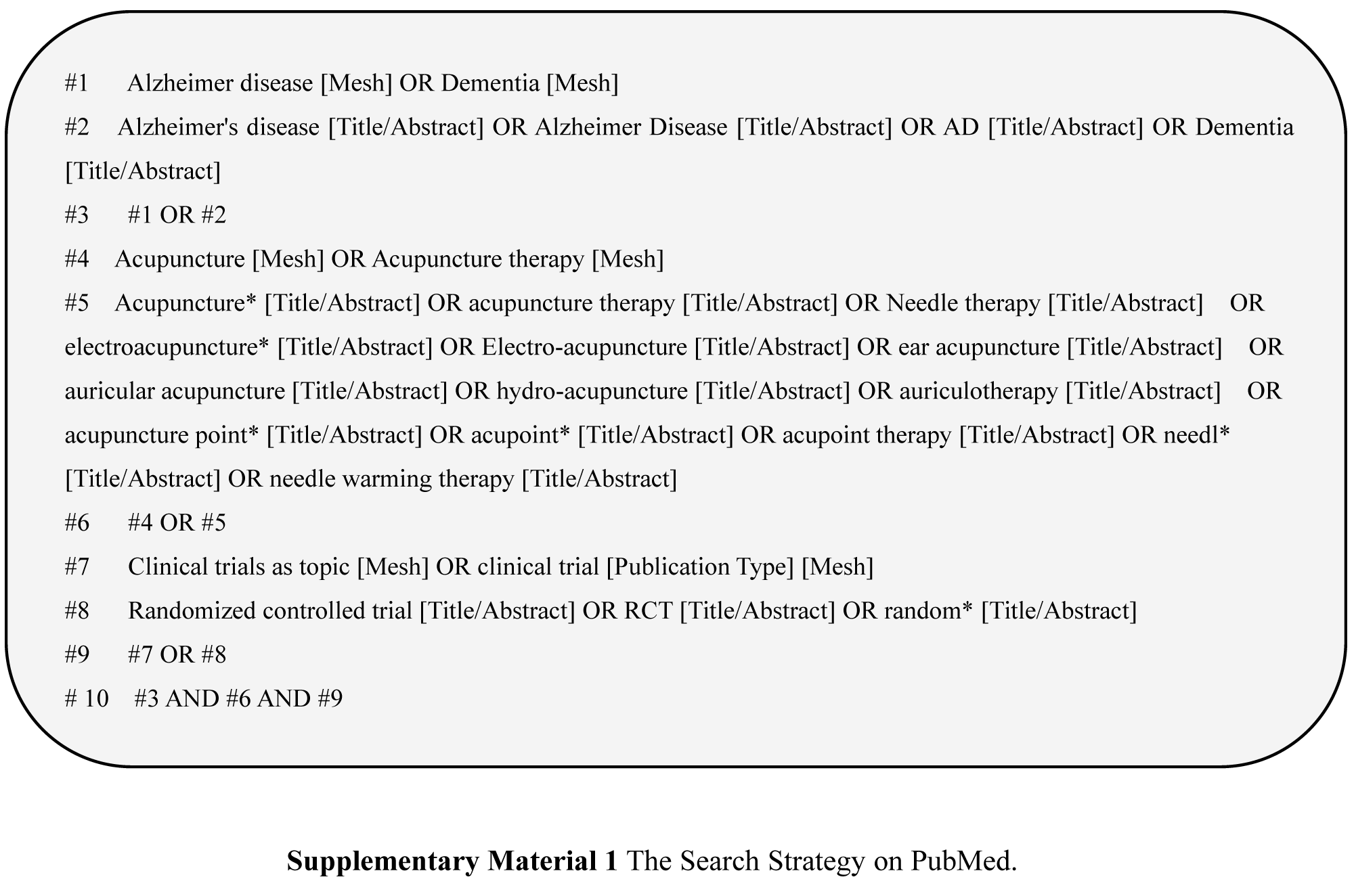

Supplement: Supplementary Data Sheet 1 — The search strategy on PubMed. [file Image_1.TIF]
